# Exacerbations and healthcare resource utilization among COPD patients in a Swedish registry-based nation-wide study

**DOI:** 10.1186/s12890-018-0573-0

**Published:** 2018-01-25

**Authors:** Gunnar Johansson, Vasili Mushnikov, Tobias Bäckström, Andreas Engström, Javaria Mona Khalid, Jennifer Wall, Fabian Hoti

**Affiliations:** 1 Department of Public Health and Caring Sciences, Family Medicine and Preventive Medicine, Uppsala University, Uppsala, Sweden; 2 EPID Research, Metsänneidonkuja 12, FI-02130 Espoo, Finland; 3 Takeda Pharmaceuticals, Stockholm, Sweden; 4 Takeda Development Centre Europe, London, UK

**Keywords:** COPD, Healthcare resource utilization, Exacerbations, Hospitalizations, Burden of disease, Chronic bronchitis, Pharmacoepidemiology

## Abstract

**Background:**

Exacerbations of chronic obstructive pulmonary disease (COPD) are an important measure of disease severity in terms of impaired disease progression, increased recovery time, healthcare resource utilization, overall morbidity and mortality. We aimed to quantify exacerbation and healthcare resource utilization rates among COPD patients in Sweden with respect to baseline treatments, exacerbation history, and comorbidities.

**Methods:**

Patients with a COPD or chronic bronchitis (CB) diagnosis in secondary care at age of ≥40 years on 1.7.2009 were identified and followed until 1.7.2010 or death. Severe exacerbations were defined as hospitalizations due to respiratory disease, and healthcare resource utilization was measured by all-cause hospitalizations and secondary care visits. Poisson regression was used adjusting for age, gender, time since COPD/CB diagnosis, and Charlson comorbidity index.

**Results:**

In 88,548 patients (54% females, mean age 72 years), previous respiratory hospitalizations and current high use of COPD medication (double or triple therapy) predicted an 8.3-fold increase in severe exacerbation rates and 1.8-fold increase in healthcare resource utilization rates in the following year, compared to patients without combination treatment and/or history of severe exacerbations.

**Conclusions:**

COPD/CB patients with history of severe exacerbations and high use of COPD medication experienced a significantly increased rate of severe exacerbations and healthcare resource utilization during the one-year follow-up.

**Electronic supplementary material:**

The online version of this article (10.1186/s12890-018-0573-0) contains supplementary material, which is available to authorized users.

## Background

By 2020, chronic obstructive pulmonary disease (COPD) is projected to become the third leading cause of death worldwide [1]. In Sweden the prevalence of COPD is believed to be between 4 and 6%, representing approximately 500,000 patients [2, 3]. It is estimated that many patients with mild COPD are undiagnosed and account for approximately 80% of the total population of patients with the disease. The remaining 20%, representing those who receive a diagnosis, are thought to suffer from a more advanced stage of COPD [3]. However, the number of patients with a new diagnosis has increased in Sweden, particularly within primary care where the use of spirometers has increased during the last decade. About 6% of the Swedish COPD patient population are considered to have a severe air-flow limitation with a forced expiratory volume in the first second (FEV1) of less than 50% of the predicted value. Patients with an advanced age and a decreasing lung function also have a higher number of comorbidities [4].

Treatment of COPD concentrates on the prevention of exacerbations of COPD, which is an important measure of disease severity in terms of negative impact on disease progression, length of recovery time, healthcare resource utilization (HRU), and overall morbidity and mortality. Based on a sample of patients in northern Sweden [5], the total cost of COPD to society has been estimated to be 1.46 billion Euros in 2010, including both direct costs due to hospitalizations and drug treatments and indirect costs resulting from loss of productivity (sick leaves and early retirement).

The pharmacological management process of COPD begins with as-needed use of inhaled short-acting beta-2 agonists (SABA) or inhaled short-acting muscarinic antagonists (SAMA) followed by one or more of long-acting beta-2 agonists (LABA) or long-acting muscarinic antagonists (LAMA) with or without inhaled glucocorticoids (ICS) [1]. For moderate to severe COPD patients, triple therapy with ICS, LABA, and LAMA has been considered the effective pharmacological choice [6, 7].

There is a need for new treatments to reduce exacerbations of COPD. On 5th July 2010 the European Commission granted marketing authorization in the EU for roflumilast, an oral selective phosphodiesterase-4 (PDE-4) inhibitor. This is the first and only PDE-4 inhibitor commercially available for COPD treatment. The aim of this Swedish registry-based study was to describe the total Swedish COPD population who had attended hospital in-patient or out-patient secondary care with respect to use of COPD medication, comorbidities, and the burden of COPD measured by exacerbations and HRU, before the introduction of roflumilast.

## Methods

In Sweden, individual-level data for all residents of the country can be linked across multiple national databases. As the aim of this study was to describe the total Swedish COPD population which had attended secondary in-patient or out–patient care with respect to medication, comorbidities, and COPD burden, data from the Centre for Epidemiology at the Swedish National Board of Health and Welfare were utilized. Patients with COPD only attending primary care were not included in this study. Briefly, the Swedish Prescribed Drug Register (filled prescriptions), the Swedish Hospital Discharge Register, the Swedish Hospital Out-Patient Register, the Swedish Death Register and the Swedish Cancer Register were used for this study. These national databases hold data on all inhabitants in Sweden and thus cover the complete population of 9.7 million inhabitants. Data from the different registers were linked by the Swedish National Board of Health and Welfare using individual patient identification (ID) and patient IDs were then pseudonymised. Ethical approval was granted by the ethics committee in Stockholm, Sweden (diary number: 2013/1412-31/2).

Living patients with a diagnosis of COPD (ICD-10 code J44) or chronic bronchitis (CB; ICD-10 code J41, J42) and aged ≥40 years on 1.7.2009 were identified from the Swedish Hospital Discharge Register and Swedish Hospital Out-Patient Register. Patients were followed from baseline (1.7.2009) for up to 1 year until 1.7.2010 or death.

Baseline medical conditions were defined based on ICD-10 diagnosis codes from in-patient hospitalizations, from 1.1.1998, and out-patient secondary care visits, from 1.1.2001. Diagnosis codes for both the primary cause and all secondary causes were utilized. Use of medication at baseline was evaluated based on specific drug purchases within the last year and within the last four months prior to baseline. This four-month period was used as a proxy for ongoing drug exposure. In Sweden, the maximum length of a reimbursed medication purchase is three months. For prior use of healthcare, the numbers of all-cause hospitalizations, secondary care out-patient visits, and hospitalizations due to respiratory diseases within one year prior to baseline were evaluated. Case-specific hospitalizations were evaluated based on the primary diagnosis only. All baseline covariates were evaluated separately for females, males, and all patients.

Study outcomes included exacerbations and HRU. Exacerbations were classified into severe and moderate exacerbations, where severe exacerbation was defined by hospitalization due to respiratory disease (ICD-10 code J09-J22 and J40-J99). Moderate exacerbations were defined as a purchase of systemic corticosteroids (ATC code H02AB) or a purchase of systemic antibiotics (ATC code J01). For the study cohort, the use of this defintion of moderate exacerbations also captured purchases that were prescribed outside of secondary care, i.e. in primary care or in the private sector. HRU was measured by all-cause hospitalizations and all-cause secondary care out-patient visits during follow-up. The distribution of exacerbations and HRU during 1 year prior to baseline was calculated separately for males, females, and all patients.

Crude rates for exacerbations and HRU were calculated within the strata of number of hospitalizations due to respiratory diseases, purchases of systemic corticosteroids, and purchases of systemic antibiotics during the previous year, and the Charlson comorbidity index (CCI). The CCI is a severity index based on comorbidities covering serious disease areas, including COPD, with the value calculated as a weighted sum of these comorbidities. More severe conditions are given a higher weight [8, 9].

COPD patients with high resource use were identified as those with at least two respiratory-related hospitalizations within 1 year prior to baseline and on-going usage of ICS combined with LABA and/or LAMA at baseline. For the high resource use COPD patient status (yes/no), a Poisson regression model was used to calculate the crude and adjusted relative ratios with 95% confidence intervals for severe exacerbations and HRU during the follow-up period. Time spent in hospital was excluded from the follow-up time. The adjusted model included the following pre-defined variables: gender, age, time since diagnosis of COPD/CB, and CCI at baseline.

In addition a Poisson model was used to quantify the contribution of the two individual components, history of respiratory hospitalizations (≥2 within 1 year) and current medication use of ICS and LABA and/or LAMA, on the risk of severe exacerbations and HRU. In this model, the binomial high resource use variable was replaced with a categorical variable with all four combinations of the two individual components represented.

## Results

A total of 88,548 patients were identified who had a diagnosis for COPD (81,070), CB (11,130), or both COPD and CB (3652). The mean age was 72.1 years (standard deviation, SD, ±10.8 years) and 53.6% of the total identified patients were female. The mean duration of disease for all patients was 4.4 years (SD ±3.2 years). Female patients were younger (difference of 0.4 years, *p* < 0.001) and had a longer duration of disease (difference of 0.2 years, *p* < 0.001) at baseline (Table 1).

**Table 1:** COPD/CB patient characteristics at baseline and medication during the year before baseline

Patient characteristics	Female *N* = 47,487 (53.6%)	Male *N* = 41,061 (46.4%)	*P*-value	Total *N* = 88,548 (100%)
Age, years	71.9 ± 11.1	72.3 ± 10.5	<0.001	72.1 ± 10.8
Time since COPD/CB diagnosis, years	4.5 ± 3.2	4.3 ± 3.2	<0.001	4.4 ± 3.2
COPD medication^a^				
SABA	1299 (2.7)	957 (2.3)	<0.001	2256 (2.5)
SAMA	1025 (2.2)	905 (2.2)	0.660	1930 (2.2)
SABA + SAMA	353 (0.7)	260 (0.6)	0.054	613 (0.7)
ICS only	3510 (7.4)	2561 (6.2)	<0.001	6071 (6.9)
LABA only	667 (1.4)	562 (1.4)	0.670	1229 (1.4)
LAMA only	2687 (5.7)	2504 (6.1)	0.006	5191 (5.9)
ICS + LABA	12,275 (25.8)	9644 (23.5)	<0.001	21,919 (24.8)
ICS + LAMA	1181 (2.5)	913 (2.2)	0.011	2094 (2.4)
LABA + LAMA	434 (0.9)	369 (0.9)	0.839	803 (0.9)
ICS + LABA + LAMA	12,197 (25.7)	9553 (23.3)	<0.001	21,750 (24.6)
No COPD medication	11,859 (25.0)	12,833 (31.3)	<0.001	24,692 (27.9)
Other medications				
Beta blockers	16,375 (34.5)	16,430 (40.0)	<0.001	32,805 (37.0)
ACE inhibitors	11,757 (24.8)	13,309 (32.4)	<0.001	25,066 (28.3)
Calcium blockers	10,423 (21.9)	8808 (21.5)	0.074	19,231 (21.7)
Angiotensin-receptor blockers	6106 (12.9)	5247 (12.8)	0.731	11,353 (12.8)
Statins	13,105 (27.6)	14,327 (34.9)	<0.001	27,432 (31.0)

Numbers presented as n (%), or mean ± SD. P-value for difference between genders

*Abbreviations*: *COPD* Chronic obstructive pulmonary disease, *CB* Chronic bronchitis, *SD* Standard deviation, *SABA* Inhaled short-acting beta-2-agonist, *SAMA* Inhaled short-acting muscarinic antagonist, *ICS* Inhaled glucocorticoids, *LABA* Long-acting beta agonist, *LAMA* Long-acting muscarinic antagonist, *ACE* Angiotensin-converting enzyme

^a^Results for SABA and/or SAMA are reported as monotherapy without any other COPD medication allowed. Use of rescue medication (SABA and/or SAMA) was ignored when evaluating usage percentages of other COPD medication options. ATC codes are listed in Additional file 1: Table S1

The baseline medication for the study population is described in Table 1. Of the total 88,548 patients, 27.9% received none of the following COPD medications either as a monotherapy or as part of a combination therapy: SABA, SAMA, ICS, LABA, or LAMA. For females, the percentage of COPD/CB patients with no COPD medication was lower compared to males (25.0% vs. 31.3%, *p* < 0.001).

For ICS, LABA, and LAMA as monotherapies, the usage percentages were 6.9%, 1.4%, and 5.9%, respectively. For use of double combination therapies, the percentage of patients on both ICS and LABA was 24.8%, while 2.4% of patients received ICS and LAMA medications, and 0.9% received LABA and LAMA. A total of 24.6% of all patients received triple combination therapy (Table 1). For mono, combo and triple therapies, the possible use of rescue medication, SABA and/or SAMA, was ignored. The use of rescue medications alone occurred in only 5.4% of the patients. For certain medications associated with comorbidities, including the use of beta blockers, angiotensin-converting enzyme (ACE) inhibitors, and statins, the percentage was higher for males compared to females (Table 1), while for calcium channel blockers and angiotensin-receptor (AR) blockers no gender difference were observed.

The distribution of comorbidities in our COPD/CB cohort is listed in Table 2. The most common comorbidities were cardiovascular disease (including coronary artery disease, congestive heart failure, atrial fibrillation, and myocardial infarction; 44.0%), hypertension (39.1%), and asthma (21.9%). The overall burden of comorbidities measured by the CCI was significantly higher for males (2.9) compared to females (2.5, *p* < 0.001).

**Table 2:** History of comorbidities and the Charlson Comorbidity Index (CCI) values at baseline

Comorbidity variable^a^	Female *N* = 47,487 (53.6%)	Male *N* = 41,061 (46.4%)	*P*-value	Total *N* = 88,548 (100%)
Cardiovascular disease	18,430 (38.8)	20,575 (50.1)	<0.001	39,005 (44.0)
Hypertension	18,519 (39.0)	16,069 (39.1)	0.683	34,588 (39.1)
Asthma	11,782 (24.8)	7593 (18.5)	<0.001	19,375 (21.9)
Any malignancy	8209 (17.3)	8325 (20.3)	<0.001	16,534 (18.7)
Diabetes	7294 (15.4)	8012 (19.5)	<0.001	15,306 (17.3)
Cerebrovascular disease	6248 (13.2)	6785 (16.5)	<0.001	13,033 (14.7)
Mood disorders	4818 (10.1)	2526 (6.2)	<0.001	7344 (8.3)
Osteoporosis	4688 (9.9)	845 (2.1)	<0.001	5533 (6.2)
Renal disease	1304 (2.7)	2074 (5.1)	<0.001	3378 (3.8)
Number of comorbidities (from those listed above)	1.71 ± 1.37	1.77 ± 1.35	<0.001	1.74 ± 1.36
0	10,233 (21.6)	8125 (19.8)		18,358 (20.7)
1	13,210 (27.8)	10,948 (26.7)		24,158 (27.3)
2	11,244 (23.7)	10,259 (25.0)		21,503 (24.3)
3-5	12,454 (26.2)	11,513 (28.0)		23,967 (27.1)
6-9	346 (0.7)	216(0.5)		562 (0.6)
CCI	2.5 ± 1.9	2.9 ± 2.1	<0.001	2.7 ± 2.0
1	18,761 (39.5)	12,850 (31.3)		31,611 (35.7)
2	10,338 (21.8)	8569 (20.9)		18,907 (21.4)
3-5	15,066 (31.7)	15,452 (37.6)		30,518 (34.5)
> 5	3322 (7.0)	4190 (10.2)		7512 (8.5)
Pneumonia/influenza^b^	2447 (5.2)	2481 (6.0)	<0.001	4928 (5.6)

Numbers presented as N (%), or mean ± SD. P-value for difference in mean values between genders

*Abbreviations*: *SD* Standard deviation, *CCI* Charlson Comorbidity Index [8, 9]

^a^Variable definitions are described at ICD-10 code level in Additional file 1: Table S1

^b^Evaluated during previous year

In total, 42.7% of the COPD/CB patients had at least one hospitalization and 71.3% had at least one secondary care out-patient visit within 1 year prior to baseline. Overall, males made more hospitalizations and secondary care out-patient visits than females (Table 3). No gender difference was observed in respiratory-related hospitalizations, where 15.2% of all patients had at least one visit. For systemic corticosteroids and systemic antibiotics, the percentage of patients with at least one purchase was 29.9% and 53.0%, respectively (Table 3). Both use of systemic corticosteroids and antibiotics, indicative of a COPD exacerbation, was higher in females compared with males.

**Table 3:** Distribution of study outcomes in COPD/CB patients during one year prior to baseline

Outcome variable	Categories	Female *N* = 47,487 (53.6%)	Male *N* = 41,061 (46.4%)	*P*-value	Total *N* = 88,548 (100%)
All cause hospitalizations		0.97 ± 1.78	1.08 ± 1.98	<0.001	1.02 ± 1.88
	0	27,540 (58.0)	23,172 (56.4)		50,712 (57.3)
	1-2	14,105 (29.7)	12,145 (29.6)		26,250 (29.6)
	3-5	4500 (9.5)	4331 (10.5)		8831 (10.0)
	>5	1342 (2.8)	1413 (3.4)		2755 (3.1)
All cause secondary care out-patient visits		2.82 ± 5.28	3.01 ± 7.45	<0.001	2.91 ± 6.38
	0	13,600 (28.6)	11,812 (28.8)		25,412 (28.7)
	1-2	16,333 (34.4)	13,941 (34.0)		30,274 (34.2)
	3-5	10,674 (22.5)	9303 (22.7)		19,977 (22.6)
	>5	6880 (14.5)	6005 (14.6)		12,885 (14.6)
Respiratory hospitalizations		0.27 ± 0.89	0.27 ± 0.87	0.273	0.27 ± 0.88
	0	40,224 (84.7)	34,857 (84.9)		75,081 (84.8)
	1-2	6070 (12.8)	5167 (12.6)		11,237 (12.7)
	3-5	953 (2.0)	865 (2.1)		1818 (2.1)
	>5	240 (0.5)	172 (0.4)		412(0.5)
Systemic corticosteroid purchases		1.28 ± 4.19	0.99 ± 3.34	<0.001	1.15 ± 3.82
	0	32,141 (67.7)	29,932 (72.9)		62,073 (70.1)
	1-2	8935 (18.8)	6530 (15.9)		15,465 (17.5)
	3-5	4112 (8.7)	3024 (7.4)		7136 (8.1)
	>5	2299 (4.8)	1575 (3.8)		3874 (4.4)
Systemic antibiotic purchases		1.60 ± 2.90	1.24 ± 2.34	<0.001	1.44 ± 2.66
	0	20,827 (43.9)	20,833 (50.7)		41,660 (47.0)
	1-2	17,029 (35.9)	13,867 (33.8)		30,896 (34.9)
	3-5	6814 (14.3)	4716 (11.5)		11,530 (13.0)
	>5	2817 (5.9)	1645 (4.0)		4462 (5.0)

Numbers presented as N (%) or as mean ± SD. *P*-value for difference in mean values between genders

*Abbreviation*: *SD* Standard deviation

**Fig. 1:**
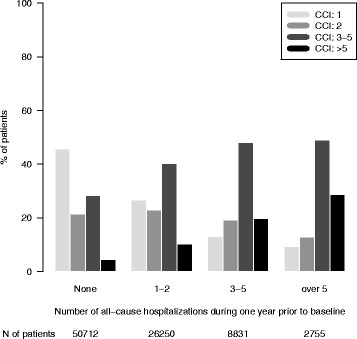
The distribution of the Charlson comorbidity index (CCI) stratified by number of all-cause hospitalizations during one year prior to baseline. The number of patients within each stratum is given in the bottom panel

**Fig. 2:**
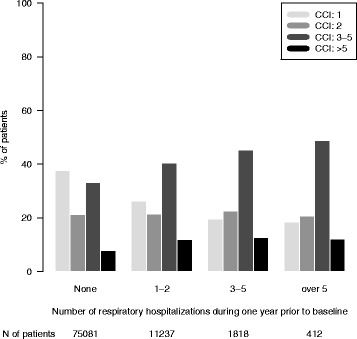
The distribution of the Charlson comorbidity index (CCI) stratified by number of respiratory hospitalizations during one year prior to baseline. The number of patients within each stratum is given in the bottom panel

Respiratory-related hospitalizations in the prior year were strong predictors of future moderate and severe exacerbations (Table 4). Patients with over five respiratory-related hospitalizations had a 37-fold higher severe exacerbation rate compared to the group with no prior respiratory hospitalizations. Similarly, prior respiratory hospitalizations predicted increased use of systemic corticosteroids and antibiotics. Figures 1 and 2 present the CCI distribution stratified by the number of all-cause hospitalizations and respiratory related hospitalizations. Patients with a higher number of hospitalizations had a higher comorbidity burden measured by CCI values.

**Table 4:** Incident rates for severe exacerbations and purchases of systemic corticosteroids or antibiotics during follow-up stratified by respiratory hospitalizations in history

Stratifying variable: Number of respiratory hospitalizations in history^a^	Severe exacerbations	Systemic corticosteroids	Systemic antibiotics
0	0.15 (0.15-0.16)	1.05 (1.04-1.05)	1.26 (1.25-1.27)
1-2	0.74 (0.72-0.76)	2.48 (2.45-2.51)	2.03 (2.01-2.06)
3-5	2.29 (2.22-2.37)	4.57 (4.47-4.68)	3.18 (3.09-3.28)
>5	5.56(5.29-5.83)	8.38 (8.05-8.72)	4.53 (4.29-4.78)

^a^One year prior to follow-up. Incidence rates presented by mean value and 95% confidence interval

Of the COPD/CB patients, 3057 (3.5%) patients were classified into the high resource use group. The high resource use COPD patient status predicted an 8.3-fold increase in the adjusted severe exacerbation rate and a 1.8-fold increase in the adjusted HRU rate during the following year, compared with patients not in the severe COPD group (Table 5).

**Table 5:** Severe exacerbations and healthcare resource utilizations among COPD patients stratified by high resource use status

High resource use^a^	N of patients	Person years	N of events	Crude rates	Crude RR	Adjusted^b^ RR
Severe exacerbations						
No	85,491	80,252	17,863	0.22	ref.	ref.
Yes	3057	2521	5293	2.10	9.43	8.31
Healthcare resource utilization						
No	85,491	80,252	310,523	3.87	ref.	ref.
Yes	3057	2521	18,976	7.53	1.95	1.82

*Abbreviation*: *RR* Relative ratio

^a^Binomial variable defined by history of respiratory hospitalizations (≥2 within 1 year) and current medication use of ICS and LABA and/or LAMA (within 4 months). Categories (Yes/No)

^b^Adjusted for age, gender, time since diagnosis, and Charlson’s comorbidity index

The increased risk of severe exacerbations and HRU use in the high resource use COPD group is due to both of the two individual components used in defining the severe COPD group (Table 6). The effect of history of respiratory hospitalizations (≥2 within 1 year) is stronger than that of current medication use of ICS and LABA and/or LAMA. When combining the two conditions, the increase of the risk is not multiplicative, implicating that the two components are correlated (Table 6).

**Table 6:** Severe exacerbations and healthcare resource utilizations stratified in COPD patients stratified by resource use status with 4 categories

High resource use^a^	N of patients	Person years	N of events	Crude rates	Crude RR	Adjusted^b^ RR
Severe exacerbations						
No, No	51,384	48,270	6764	0.14	ref.	ref.
No, Yes Yes, No Yes, Yes	32,269 1838 3057	30,516 1465 2521	8758 2341 5293	0.29 1.60 2.10	2.05 11.40 14.98	2.07 10.35 13.46
Healthcare resource utilization						
No, No	51,384	48,270	179,149	3.71	ref.	ref.
No, Yes Yes, No Yes, Yes	32,269 1838 3057	30,516 1465 2521	119,372 12,002 18,976	3.91 8.19 7.53	1.05 2.21 2.03	1.12 1.89 1.95

*Abbreviation*: *RR* Relative ratio

^a^Categorical variable with 4 combinations of the following two variables: history of respiratory hospitalizations (≥2 within 1 year), and current medication use of ICS and LABA and/or LAMA (within 4 months). Categories (No/No, No/Yes, Yes/No, Yes/Yes)

^b^Adjusted for age, gender, time since diagnosis, and Charlson comorbidity index

## Discussion

We identified 88,548 patients with a hospital-based diagnosis of either COPD or CB prior to baseline. Of all the patients, 72.1% used at least one COPD medication and the majority on a daily basis during the previous year. The most common treatments were a combination therapy with ICS and LABA (24.8%) or triple therapy (24.6%). These most severe patients had substantially more exacerbations and used more health care resources even though they were prescribed most of the medications indicated for treatment of COPD.

High-dose ICS are widely used in the management of COPD, either alone or combined with a LABA. ICS can improve health status and reduce exacerbation rates but ICS, even in high doses, fail to suppress inflammation in COPD lungs and airways [4, 10]. LABAs are useful bronchodilators in COPD patients, but it is uncertain whether they have anti-inflammatory effects [11, 12].

LABA and LAMA are effective treatments for the moderate to severe COPD patients and result in a reduction of exacerbations. Concurrent administration of a LABA with a LAMA produces superior bronchodilation compared with their individual effects. Inhaled corticosteroid therapy in combination with LABA have been shown to be of greater benefit than ICS alone [13–16]. Interesting, 7% of the patients are using only ICS even though ICS as a monotherapy, without LABA or LAMA, is not indicated for COPD. However, as 22% of the patients also had asthma it may be that asthma is the main reason for the ICS treatment.

For moderate to severe COPD patients, triple therapy is the effective pharmacological choice [6, 7] resulting in improved lung function, reduction of exacerbations, and an improved quality of life in comparison to monotherapy [17]. In our study population, which cover all Swedish patients with COPD who have attended secondary care, half of the patients are treated with ICS and LABA or triple therapy.

Patients with COPD tend to carry a heavy burden of comorbidities [4, 18, 19], which is also in agreement with the results from our study. The most prevalent comorbidities are cardiovascular and cerebrovascular disease in both our study and other studies [4, 18, 19]. For cardiovascular disease the prevalence in males was higher (50.1%) compared to females (38.8%) and more than one third of all patients had a diagnosis of hypertension. For other comorbidities including any malignancy, diabetes, cerebrovascular disease, and renal disease, the prevalence was also higher in males compared to females. In contrast, the prevalence of asthma, mood disorders, and osteoporosis were much higher in females compared to males. One potential source of bias is that patients with an asthma diagnosis may have been wrongly diagnosed as COPD patients. To see if this would impact our results we rerun the Poisson models in Tables 5 and 6 after excluding patients with an asthma diagnosis. The results did not change (see Additional file 1: Tables S2 & S3).

Another interesting observation is that the average number of hospitalizations for any reason was one per patient during the last year compared with 0.3 hospitalizations for respiratory reasons, indicating how severe these patients are with a high burden of comorbidities. Another indicator of the impact of the comorbidities is the finding of the strong correlation between the Charlson comorbidity index and the all-cause and respiratory related hospitalization rates.

Of 88,548 COPD patients, 3057 (3.5%) were classified into the high resource use group. These patients had an 8.3-fold increased risk of a new severe exacerbation compared to the other COPD patients during the follow-up year. Even though these patients used most of the available treatments for COPD they would probably benefit from additional effective treatment in order to reduce their risk of exacerbation. Other options to reduce progress of the disease is non-pharmaceutical interventions or a diagnosis of COPD at an earlier stage. However, with the present design of our study with data from different national healthcare registers it is not possible to obtain this information.

One of the limitations of this study is that COPD patients without in- or out-patient hospitalizations are not taken into account. Patients with only primary care visits is most probably a healthier population, whereas the hospitalized individuals are a more severe group of COPD patients who are more likely to have moderate or severe exacerbations. The target population for alternative therapies is not in the primary care group however, but the more severe patients with frequent exacerbations and hospitalizations. Another limitation that applies to the definition of moderate exacerbation is that the reason for prescribing systemic corticosteroid or systemic antibiotic is not available, therefore the estimated moderate exacerbation rates may be overestimates.

Despite a lack of primary care patients included in this study, another limitation with this study population is the diversity of patients, with 28% of the patients having no medication for COPD despite a diagnosis of COPD and 5% using only short-acting bronchodilators. There may be several reasons for untreated mild COPD patients to obtain a hospital-based diagnosis. The patients may have been admitted to secondary out-patient care to be investigated concerning COPD and thus after this investigation have achieved the COPD diagnosis, but with no or limited COPD medication. Similarly, the patient may have been admitted to hospital for another reason but when discharged from the hospital they were also given a diagnosis of mild COPD. Finally, a further potential limitation with this study is the lack of data on lung function (spirometry values/grades), an important measure of COPD severity. However, such data was not available in the utilized national registers.

There are a number of important strengths in this study. The large number of patients with COPD and in most cases with an advanced disease representing the Swedish patients attending secondary care. The real-world design reflects the normal pharmaceutical therapy care of these COPD patients as well as describes their use of secondary care in the whole Sweden.

This paper describes a nation-wide register based study of COPD/CB patients with a varying history of severe exacerbations and medication use. COPD patients with high resource use continue to experience significantly increased rates of severe exacerbations and use of healthcare resources, indicating a potential unmet need in this group of patients. The need for new pharmaceutical therapies with the aim to reduce severe exacerbations is evident and may in the future be of benefit for this high risk population.

## Conclusions

In conclusion, findings from this study suggest that COPD/CB patients with a history of severe exacerbations as well as a high use of COPD medication experienced a significantly increased rate of continued severe exacerbations and healthcare resource utilization compared to those without. These findings indicate a potential unmet need for new therapies in a high risk population of patients.

## Additional file

Additional file 1: Supplementary information. (DOC 96 kb)

## Abbreviations

ACE: Angiotensin-converting enzyme
AR: Angiotensin-receptor
ATC: Anatomical therapeutic chemical
CB: Chronic bronchitis
CCI: Charlson comorbidity index
COPD: Chronic obstructive pulmonary disease
HRU: Healthcare resource utilization
ICD-10: 10th revision of the international classification of diseases
ICS: Inhaled glucocorticoids
LABA: Long-acting beta-2 agonists
LAMA: Long-acting muscarinic antagonists
MI: Myocardial infarction
PDE-4: Phosphodiesterase-4
SABA: Short-acting beta-2 agonists
SAMA: Short-acting muscarinic antagonists
SD: Standard deviation
