# Supplementary material for: Exacerbations and healthcare resource utilization among COPD patients in a Swedish registry-based nation-wide study
Source: BMC Pulm Med. 2018 Jan 25;18:17. doi: 10.1186/s12890-018-0573-0 (PMC5784707; doi:10.1186/s12890-018-0573-0)
Supplement: Additional file 1: — Supplementary information. (DOC 96 kb) [file 12890_2018_573_MOESM1_ESM.doc]

| **Table S1:** Study variable definitions | | |
| --- | --- | --- |
| **Variable** | **Description** | **Operational definition:**  **ATC codes/ICD-10 codes** |
| Gender | Male/Female |  |
| Age | Age on 1.7.2009, classified as 40-44, 45-49, 50-54, 55-59, 60-64, 65-69, 70-74, 75-79, ≥80 |  |
| Time since diagnosis | Time interval (years) between the first COPD/CB diagnose and 1.7.2009 |  |
| SABA use | Purchase of SABA within the last year prior to 1.7.2009 | ATC: R03AC02, R03AC03 |
| SAMA use | Purchase of SAMA within the last year prior to 1.7.2009 | ATC: R03BB01 |
| SAMA+SABA use | Purchase of SAMA+SABA within the last year prior to 1.7.2009 | ATC: R03BB01+R03AC02, R03BB01+R03AC03 |
| ICS use | Purchase of ICS within the last year prior to 1.7.2009 | ATC: R03BA |
| LAMA use | Purchase of LAMA within the last year prior to 1.7.2009 | ATC: R03BB04 |
| LABA use | Purchase of LABA within the last year prior to 1.7.2009 | ATC: R03AC12, R03AC13 |
| ICS+LAMA use | Purchase of ICS+LAMA within the last year prior to 1.7.2009 | ATC: R03BA+R03BB04 |
| ICS+LABA use | Purchase of ICS+LABA within the last year prior to 1.7.2009 | ATC: R03AK06, R03AK07, R03BA+R03AC12, R03BA+R03AC13 |
| LAMA+LABA use | Purchase of LAMA+LABA within the last year prior to 1.7.2009 | ATC: R03BB04+R03AC12, R03BB04+R03AC13 |
| ICS+LABA+LAMA use | Purchase of ICS+LABA+LAMA within the last year prior to 1.7.2009 | ATC: R03AK06+R03BB04, R03AK07+R03BB04, R03BA+R03AC12+R03BB04, R03BA+R03AC13+R03BB04 |
| Current medication use of ICS+LABA and/or LAMA | Purchase of ICS+LABA and/or LAMA within 4 months prior to 1.7.2009 | ATC: R03BA+R03BB04, R03AK06, R03AK07, R03BA+R03AC12, R03BA+R03AC13, R03AK06+R03BB04, R03AK07+R03BB04, R03BA+R03AC12+R03BB04, R03BA+R03AC13+R03BB04 |
| Beta blocker use | Purchase of beta blockers within the last year prior to 1.7.2009 | ATC: C07 |
| ACE inhibitor use | Purchase of ACE inhibitors within the last year prior to 1.7.2009 | ATC: C09 excluding C09CA |
| Calcium channel blocker use | Purchase of calcium channel blockers within the last year prior to 1.7.2009 | ATC: C08 |
| AR blocker use | Purchase of AR blockers within the last year prior to 1.7.2009 | ATC: C09CA |
| Statin use | Purchase of statins within the last year prior to 1.7.2009 | ATC: C10AA |
| N of systemic antibiotic purchases | Number of reimbursed purchase episodes for systemic antibiotics within the last year prior to 1.7.2009 | ATC: J01 |
| N of systemic corticosteroid purchases | Number of reimbursed purchase episodes for systemic corticosteroids within the last year prior to 1.7.2009 | ATC: H02AB |
| N of all-cause hospitalizations | Number of hospitalizations for any cause within the last year prior to 1.7.2009 |  |
| N of secondary care visits | Number of secondary care outpatient visits within the last year prior to 1.7.2009 |  |
| N of respiratory hospitalizations | Number of respiratory hospitalizations within the last year prior to 1.7.2009 | ICD-10: J09-22, J40-J99 |
| Pneumonia/influenza | Occurrence of pneumonia/influenza within 1 year prior to 1.7.2009 (Yes/No) | ICD-10: J09-J18 |
| Cardiovascular diseases | History of cardiovascular diseases up to 1.7.2009 | Includes coronary heart disease, congestive heart failure, atrial fibrillation and myocardial infarction |
| Coronary heart disease | History of coronary heart disease (other than MI) up to 1.7.2009 | ICD-10: I20, I24, I25, excluding I25.2 for MI |
| Congestive heart failure | History of congestive heart failure up to 1.7.2009 (Yes/No) | ICD-10: I09.9, I11.0, I13.0, I13.2, I25.5, I42.0, I42.5-I42.9, I43.x, I50.x, P29.0 |
| Atrial fibrillation | History of atrial fibrillation up to 1.7.2009 (Yes/No) | ICD-10: I48.0-I48.2, I48.9 |
| Myocardial infarction | History of myocardial infarction up to 1.7.2009 (Yes/No) | ICD-10: I21, I22, I25.2 |
| Hypertension | History of hypertension up to 1.7.2009 (Yes/No) | ICD-10: I10-I15 |
| Asthma | History of bronchial asthma diagnose up to 1.7.2009 (Yes/No) | ICD-10: J45 |
| Any malignancy | History of malignancy diagnose (including lymphoma and leukemia, except malignant neoplasm of skin) up to 1.7.2009 (Yes/No) | ICD-10: C00-C26, C30-C34, C37-C41, C43, C45-C58, C60-C76, C81-C85, C88, C90-C97 |
| Diabetes | History of diabetes up to 1.7.2009 (Yes/No) | ICD-10: E10-E14, ATC: A10 |
| Cerebrovascular disease | History of cerebrovascular disease up to 1.7.2009 (Yes/No) | ICD-10: G45, G46, H34.0, I60-I69 |
| Mood disorder | History of mood disorder up to 1.7.2009 (Yes/No) | ICD-10: F30-F39 |
| Osteoporosis | History of osteoporosis up to 1.7.2009 (Yes/No) | ICD-10: M80-M82 |
| Renal disease | History of renal disease up to 1.7.2009 (Yes/No) | ICD-10: I12.0, I13.1, N03.2-N03.7, N05.2-N05.7, N18, N19, N25.0, Z49.0-Z49.2, Z94.0, Z99.2 |
| Number of comorbidities | Number of comorbidities up to 1.7.2009 | Counts include cardiovascular disease, hypertension, asthma, any malignancy, diabetes, cerebrovascular disease, mood disorder, osteoporosis, renal disease |
| CCI | For whole history up to 1.7.2009. Categories: 0, 1, 2, 3-5, >5 |  |
| Abbreviations: ATC, Anatomical Therapeutic Chemical; ICD-10, 10^th^ revision of the International Classification of Diseases; COPD, chronic obstructive pulmonary disease; CB, chromic bronchitis; SABA, inhaled short-acting beta-2 agonist; SAMA, inhaled short-acting muscarinic antagonist; ICS, inhaled glucocorticoids; LAMA, long-acting muscarinic antagonist; LABA, long-acting beta-2 agonist; ACE, angiotensin-converting enzyme; AR, angiotensin-receptor; MI, myocardial infarction; CCI, Charlson comorbidity index | | |

| **Table S2:** Severe exacerbations and healthcare resource utilizations among COPD patients stratified by high resource use status*. Patients with an Asthma diagnose are excluded.* | | | | | | |
| --- | --- | --- | --- | --- | --- | --- |
| **High resource use^1^** | **N of patients** | **Person years** | **N of events** | **Crude rates** | **Crude RR** | **Adjusted ^2^ RR** |
|  | **Severe exacerbations** | | | | | |
| No | 67031 | 62858 | 13474 | 0.21 | ref. | ref. |
| Yes | 2142 | 1756 | 3613 | 2.06 | 9.60 | 8.57 |
|  | **Healthcare resource utilization** | | | | | |
| No | 67031 | 62858 | 234194 | 3.73 | ref. | ref. |
| Yes | 2142 | 1756 | 12620 | 7.19 | 1.93 | 1.83 |
| Abbreviation: RR, relative ratio  ^1^ Binomial variable defined by history of respiratory hospitalizations (≥2 within 1 year) and current medication use of ICS and LABA and/or LAMA (within 4 months). Categories (Yes/No)  ^2^ Adjusted for age, gender, time since diagnosis, and Charlson’s comorbidity index | | | | | | |
| | **Table S3:** Severe exacerbations and healthcare resource utilizations stratified in COPD patients stratified by resource use status with 4 categories.*. Patients with an Asthma diagnose are excluded.* | | | | | | | | --- | --- | --- | --- | --- | --- | --- | | **High resource use^1^** | **N of patients** | **Person years** | **N of events** | **Crude rates** | **Crude RR** | **Adjusted ^2^ RR** | |  | **Severe exacerbations** | | | | | | | No, No | 42 830 | 40 247 | 5 408 | 0.13 | ref. | ref. | | No, Yes  Yes, No  Yes, Yes | 22 818  1 383  2 142 | 21 515  1 097  1 756 | 6 467  1 599  3 613 | 0.30  1.46  2.06 | 2.24  10.85  15.31 | 2.25  9.98  13.87 | |  | **Healthcare resource utilization** | | | | | | | No, No | 42 830 | 40 247 | 145 370 | 3.61 | ref. | ref. | | No, Yes  Yes, No  Yes, Yes | 22 818  1 383  2 142 | 21 515  1 097  1 756 | 80 435  8 389  12 620 | 3.74  7.65  7.19 | 1.04  2.12  1.99 | 1.11  1.80  1.93 | | Abbreviation: RR, relative ratio  ^1^ Categorical variable with 4 combinations of the following two variables: history of respiratory hospitalizations (≥2 within 1 year), and current medication use of ICS and LABA and/or LAMA (within 4 months). Categories (No/No, No/Yes, Yes/No, Yes/Yes)  ^2^ Adjusted for age, gender, time since diagnosis, and Charlson comorbidity index | | | | | | | | | | | | | |
